# *S*-Palmitoylation as a Functional Regulator of Proteins Associated with Cisplatin Resistance in Bladder Cancer

**DOI:** 10.7150/ijbs.45640

**Published:** 2020-07-19

**Authors:** Muhammad Shahid, Minhyung Kim, Peng Jin, Bo Zhou, Yang Wang, Wei Yang, Sungyong You, Jayoung Kim

**Affiliations:** 1 Departments of Surgery and Biomedical Sciences, Cedars-Sinai Medical Center, Los Angeles, CA, USA.; 2 Department of Medicine, University of California Los Angeles, CA 90095, USA.

**Keywords:** *S*‐palmitoylation, lipid, lipidation, post‐translational modification, tumor

## Abstract

Protein *S*-palmitoylation is a powerful post-translational modification that regulates protein trafficking, localization, turnover, and signal transduction. Palmitoylation controls several important cellular processes, and, if dysregulated, can lead to cancer, cardiovascular disease, and neurological disorders. The role of protein palmitoylation in mediating resistance to systemic cisplatin-based chemotherapies in cancer is currently unknown. This is of particular interest because cisplatin is currently the gold standard of treatment for bladder cancer (BC), and there are no feasible options after resistance is acquired. Using unbiased global proteomic profiling of purified *S*-palmitoylated peptides combined with intensive bioinformatics analyses, we identified 506 candidate palmitoylated proteins significantly enriched in cisplatin-resistant BC cells. One of these proteins included PD-L1, which is highly palmitoylated in resistant cells. Pharmacological inhibition of fatty acid synthase (FASN) suppressed PD-L1 palmitoylation and expression, which suggests the potential use of FASN-PD-L1-targeted therapeutic strategies in BC patients. Taken together, these results highlight the role of protein palmitoylation in mediating BC chemoresistance.

## Introduction

Systemic cisplatin-based chemotherapy is the current gold standard of treatment for metastatic bladder cancer (BC) 1-3. However, acquired chemoresistance is common; thereby, limiting the usage of cisplatin. Patients who acquire chemoresistance ultimately have no further viable treatment options and their cancer usually recurs. The 5-year survival rate for BC after recurrence is approximately 15% 4-6. As of now, there is a lack of comprehensive understanding behind the mechanisms driving cisplatin resistance. Thus, there is an unsolved and urgent need to identify a method of distinguishing which BC patients are at a higher risk of developing chemoresistance.

Metabolic reprogramming has been accepted as a hallmark of cancer 7-10. These changes in metabolic activities may also be involved in cisplatin-induced cell death. As a result, metabolic studies on cisplatin resistance may potentially lead to new clues for improving therapies against refractory BC. Several prior metabolomic studies found that BC may have abnormalities in metabolites involved in lipid usage 11,12. It has also been suggested that perturbed metabolism may be implicated in cancer drug-resistance, aggressiveness, or progression 13. Consistent with these earlier findings, our recent study suggested that alterations in acetate and lipid metabolism, which is mediated by acetyl-CoA synthetase 2 (ACSS2), an enzyme that converts acetate to acetyl-CoA, plays a role in cisplatin resistance 14. By performing unbiased data-driven analysis, as described below, our real-time live metabolomics identified metabolic reprogramming in a series of isogenic cisplatin-sensitive and resistant BC cell lines. In a previous study, we also found that glucose-derived endogenous acetate contributes to cell viability and increased de novo synthesis of lipids via ACSS2 15. However, the role of these bioactive metabolites in influencing lipid metabolism in BC cisplatin resistance is not fully understood.

Lipid modification of proteins at the post-translational level mostly occurs on cysteine thiols through the covalent addition of long-chain fatty acids (predominantly 16-carbon palmitic acid) 16,17. This is called protein palmitoylation and can lead to an increase in hydrophobicity of cytoplasmic proteins and an affinity to cytosolic membrane surfaces 18. Palmitoylation is an attractive mechanism for modulating protein activity, stability, interactions, localization, signaling transduction, apoptosis, and carcinogenesis 19-24. It has been shown that palmitoylation is particularly important for protein stability; it suppresses degradation by preventing ubiquitylation 19. The role of palmitoylation in protein stability has been demonstrated in a wide variety of targets and diseases, including c-Met, TEAD transcription factor, progressive rod-cone degeneration, and Huntington's disease 16,25-27. Based on the critical role of palmitoylation in protein function and disease, investigating its impact on acquired chemoresistance in BC presents a promising opportunity. From our original palmitoyl-protein identification and site characterization (PalmPISC) method 28, we previously developed a low-background acyl-biotinyl exchange (LB-ABE), a significantly improved method for successful purification and identification of hydrophobic palmitoylated proteins 28,29. By largely eliminating the co-isolation of non-palmitoylated proteins, LB-ABE minimizes the “ratio compression” issue and substantially improves quantification accuracy 29,30.

Fatty acid synthase (FASN) is a multifunctional enzyme that is involved in the de-novo synthesis of lipids 31. Its main function is catalyzing the synthesis of 16-C palmitate 32. Overexpression of FASN has been noted in a variety of different tumor types, including non-muscle invasive BC (NMIBC), and is significantly associated with poorer prognoses 33. Studies have shown that FASN is a particularly informative prognostic predictor in BC; expression was found to be positively correlated with tumor aggressiveness, histologic grade, recurrence, and poor survivability in patient cohorts 34,35. Additionally, inhibition of FASN via siRNA increased apoptosis and decreased proliferation in BC cells 36. The primary product of FASN, palmitate, plays an especially important in protein palmitoylation by attaching to and regulating protein localization, stability, and function 17. Consequently, FASN is directly involved in the palmitoylation of proteins.

Current understanding of protein palmitoylation (more accurately termed *S*-acylation) status and its role in cisplatin resistance remains very limited. In this paper, our proteome-scale analysis of adipocyte *S*-acylated proteins in cisplatin-sensitive vs resistant BC cells suggest 506 putative palmitoylated proteins associated with cisplatin resistance. We also tested the hypothesis that lipid metabolism changes in cisplatin-resistant BC cells may be mediated by the S-palmitoylation of FASN. Our experimental results demonstrated that protein palmitoylation of FASN contributes to cisplatin resistance in BC cells. This study also provides evidence suggesting that FASN inhibition alters the palmitoylation of programmed death ligand-1 (PD-L1).

## Materials and Methods

### Cell culture

Parental T24 human BC cells were procured from American Type Culture Collection. Cisplatin-sensitive (T24S) and resistant T24 (T24R) BC cells were developed and characterized in the laboratory 37. Cells were cultured in Dulbecco's modified eagle medium (DMEM) supplemented with 10% fetal bovine serum, 2% glutamine, and 1% antibiotics (Invitrogen, Carlsbad, CA). All BC cells used for this paper were maintained under a humidified atmosphere of 5% CO_2_ at 37 °C.

### Antibodies and reagents

The following antibodies and dilutions were used according to manufacturer's instructions: β-actin (A1978) from Sigma; PD-L1 (13684) (1:1000), FASN (3180) (1:1000) and HRP-conjugated secondary antibodies, rabbit (7074) (1:3000), mouse (7076) (1:3000), from Cell Signaling Technology.

### Palmitoyl-protein enrichment using LB-ABE

Palmitoyl proteins were enriched using our LB-ABE method^30^. Briefly, after cell lysis, 0.7 mg of protein from each replicate was reduced by 50 mM tris(2-carboxyethyl)phosphine (TCEP) and alkylated sequentially by 50 mM N-ethylmaleimide and 50 mM 2,2'-dithiodipyridine (DTDP). Palmitoyl proteins were converted into biotinylated proteins using 2 M neutral hydroxylamine and 1 mM biotin-N-[6-(biotinamido) hexyl]-3'-(2'-pyridyldithio) propionamide (HPDP), enriched by streptavidin affinity purification, and eluted by 50 mM TCEP.

### CAPTUREome™ S-Palmitoylated Protein Kit Assay

Confirmation of S-palmitoylated proteins in BC cells was conducted using the commercially available CAPTUREome™ S-Palmitoylated Protein Kit (cat # K010-311, Badrilla, UK). Following the indicated 2-bromopalmitate (2-BP) treatments, cells were collected and washed in phosphate-buffered saline (PBS). The methodology for acyl-RAC, including blocking of free thiols, cleavage of thioester linkages, and capture of nascent thiols on Sepharose, was carried out according to the manufacturer's instructions. In particular, equal amounts of protein (1-2 mg) were diluted in 500 μl of blocking buffer (buffer A and thiol blocking reagent) and incubated at 40 °C for 4 hours with constant shaking. Three volumes of cold acetone were added, and proteins were allowed to precipitate at -20°C for 20 min. Following centrifugation of the solution at 16,000 g for 5 min, the pellet was extensively washed with 70% acetone five times and air-dried completely after the final wash. Pellet was re-dissolved in 300 μl binding buffer and incubated in a shaking heat block at 40°C for 1 hour. The homogenates were centrifuged at 16,000 × g for 5 min to remove insoluble debris. Approximately 20 μl of each supernatant was saved as the “total input.” The pre-washed capture resin slurry (50 μl) was added to the remaining lysates, and 19 μl of thioester cleavage reagent was then added. Binding reactions were carried out on a rotator at room temperature for 2.5 hours. Resins were washed at least five times with binding buffer. Supernatants were removed and mixed with 2× Laemmli loading buffer, heated to 60 °C for 10 min, and separated via SDS-PAGE. All kits were used following the manufacturer's instructions.

### Palmitoyl protein digestion and label-free proteomic analysis

Enriched palmitoyl proteins were digested by trypsin, using filter-aided sample preparation (FASP) 38,39. Label-free proteomic analysis was performed using an EASY-nLC 1000 connected to an LTQ Orbitrap Elite Hybrid Mass Spectrometer, as previously described 29. Tryptic peptides were loaded onto a 2 cm trap column and separated on a 50 cm EASY-Spray Analytical Column heated to 55 °C, using a gradient of 2-34% B in 174 min, 34-60% B in 10 min, 60-100% B in 2 min, and 100% B in 14 min at the flow rate of 150 nL/min. Mass spectra were acquired in a data-dependent manner, with automatic switching between mass spectrometry (MS) and tandem mass spectrometry (MS/MS) scans. In the MS scans, a lock mass at m/z 445.120025 was applied to provide internal mass calibration. The full scan was performed using a 240,000 resolution at m/z 400 Th, with an ion packet setting of 1×10^6^ for automatic gain control and maximum injection time of 500 ms. The 20 most intense peptide ions with charge state ≥2 were automatically selected for MS/MS fragmentation by rapid collision-induced dissociation (rCID), using a resolution of 7,500, 1×10^4^ automatic gain control, 50 ms maximum injection time, 10 ms activation time, and 35% normalized collision energy. Dynamic exclusion was enabled with a repeat count of 1, an exclusion duration of 30 s, and a repeat duration of 90 s.

### Database searching for protein identification and quantification

The acquired MS data was searched against the Uniprot_Human Database (released on 01/22/2016, containing 20,985 sequences) using the Andromeda 40 algorithm in the MaxQuant 41 (v1.5.5.1) environment. The searching parameters were set as follows: trypsin/P as the protease; oxidation (M), acetyl (protein N-term), NEM(C), and carbamidomethyl (C) as variable modifications; up to two missed cleavages; minimal peptide length as 7; mass tolerance for MS1 was 4.5 ppm for main search and for MS2 was 0.5 Da; identification of second peptides enabled; label-free quantification (LFQ) enabled, and match-between-runs within 2 min were enabled. A stringent 1% FDR was used to filter PSM, peptide, and protein identifications.

### Identification of differentially palmitoylated proteins (DPPs)

LFQ intensities were normalized using the quantile normalization method 42 to compare different conditions. To filter out low-quality proteins, selected proteins were detected in at least two samples under each condition. DPPs between T24S and T24R cells were identified using a previously reported statistical test 43. Briefly, log_2-_intensities of each protein from T24R cells were compared to those in T24S cells using the Student's t-test and log_2_-median ratio test. We estimated empirical null distributions of t-values and log2-median ratio values by randomly permuting 6 samples 1,000 times and calculating the t-test and log2-median ratio test. These two p-values were then integrated into an overall p-value using Stouffer's method 44. DPPs were identified as proteins with overall P<0.05 and absolute log_2_-fold-change ≥ 0.58.

### Functional enrichment analysis

Enrichment analysis of gene ontology biological process (GOBPs) and Kyoto Encyclopedia of Genes and Genomes (KEGG) pathways for DPPs was performed using DAVID 45. The functional classification analysis of GOBPs, gene ontology cellular components (GOCCs), and gene ontology molecular functions (GOMFs) was performed using PANTHER (Ver. 11) 46.

### Reconstruction of a network model

To reconstruct a network model describing cisplatin resistance in BC cells, a subset of genes that are involved in metabolic processes was selected. Interaction information of the genes from the STRING database (Ver. 10.5) 47 was then collected and used to reconstruct a network model. Finally, the network model was visualized using Cytoscape 48. The nodes in the network model were distributed according to the metabolic pathways that they are involved in.

### Western blot analysis

Whole-cell lysates for western blot analysis were prepared as described in a previous paper 49. T24R and T24S BC cells were cultured on 10 cm plates and treated with 2-BP or orlistat at varying doses. Cells were lysed with RIPA buffer (20 mM Tris, 150 mM NaCl, 1% Nonidet P-40, 0.1 mM EDTA) (Pierce, ThermoFisher) supplemented with a phosphatase inhibitor cocktail (ThermoFisher), homogenized, and centrifuged at 13,000 ×g and 4°C for 20 min. Afterwards, 25 μg of protein lysates per lane was run on a 4-15% gradient SDS-PAGE gel. Following protein transfer onto polyvinylidene fluoride (PVDF), the membranes were blocked with either 5% bovine serum albumin (BSA) or 5% nonfat milk in Tris-buffered saline with 0.1% Tween 20 (TBST [2.42 g/L Tris-HCl, 8 g/L NaCl, and 1 mL/L Tween 20 (pH 7.6)]) for 1 hour at room temperature. This was followed by incubation with specific primary antibodies at 4 °C overnight and 3 X 10 min washes with TBST solution. The membranes were further incubated with HRP-conjugated secondary antibodies at room temperature for 1 hour. β-actin was used as a loading control. Experiments were performed in at least triplicates for each western blot analysis.

### Measurement of palmitate and cholesterol

Levels of palmitate in T24S and T24R cells were determined by targeted metabolomics analysis through the University of Florida Metabolomics Core using mass spectrometry-based targeted metabolomics analysis. Levels of cholesterol in the BC cells were determined using the commercially available Cholesterol Quantification Kit (cat #MAK043, Sigma, MO, USA). Cells were lysed in 1% (w/v) Triton X-100 in chloroform. The homogenates were centrifuged at 13,000 × g for 10 min to remove insoluble debris, and the organic phase (lower phase) was collected and dried in a 50°C dry bath for 20 min. Samples were vacuum dried for 30 min to remove traces of chloroform. The dried lipids were resuspended via vortex in an assay buffer and further quantified. All kits were used in accordance with the manufacturer's instructions.

### Cell proliferation assay

Cells were seeded at a density of 2×10^6^ cells/well in 12-well plates. Cells were then incubated with standard growth medium and treated with varying doses of 2-BP or orlistat, as described in the figures. Cells were stained with 0.05% crystal violet solution after removing the medium. After incubation at room temperature for 15 min, the wells were washed thoroughly with PBS, and cells were fixed with 4% paraformaldehyde at room temperature for 5 min. For quantitative analysis, the stained cells were dissolved in 10% acetic acid solution for reading absorbance at 570-590 nm 50. All experiments were run in at least triplicates.

### Statistical analysis

Student's t-tests were performed to evaluate differential expression of the proteins between two groups. Variables with normal distribution were expressed as mean ± standard deviation (SD). All reported p-values are two-tailed, with P<0.05 being considered as statistically significant.

## Results

### Palmitoyl-proteomics analysis identified DPPs associated with cisplatin resistance in BC cells

**Figure 1:**
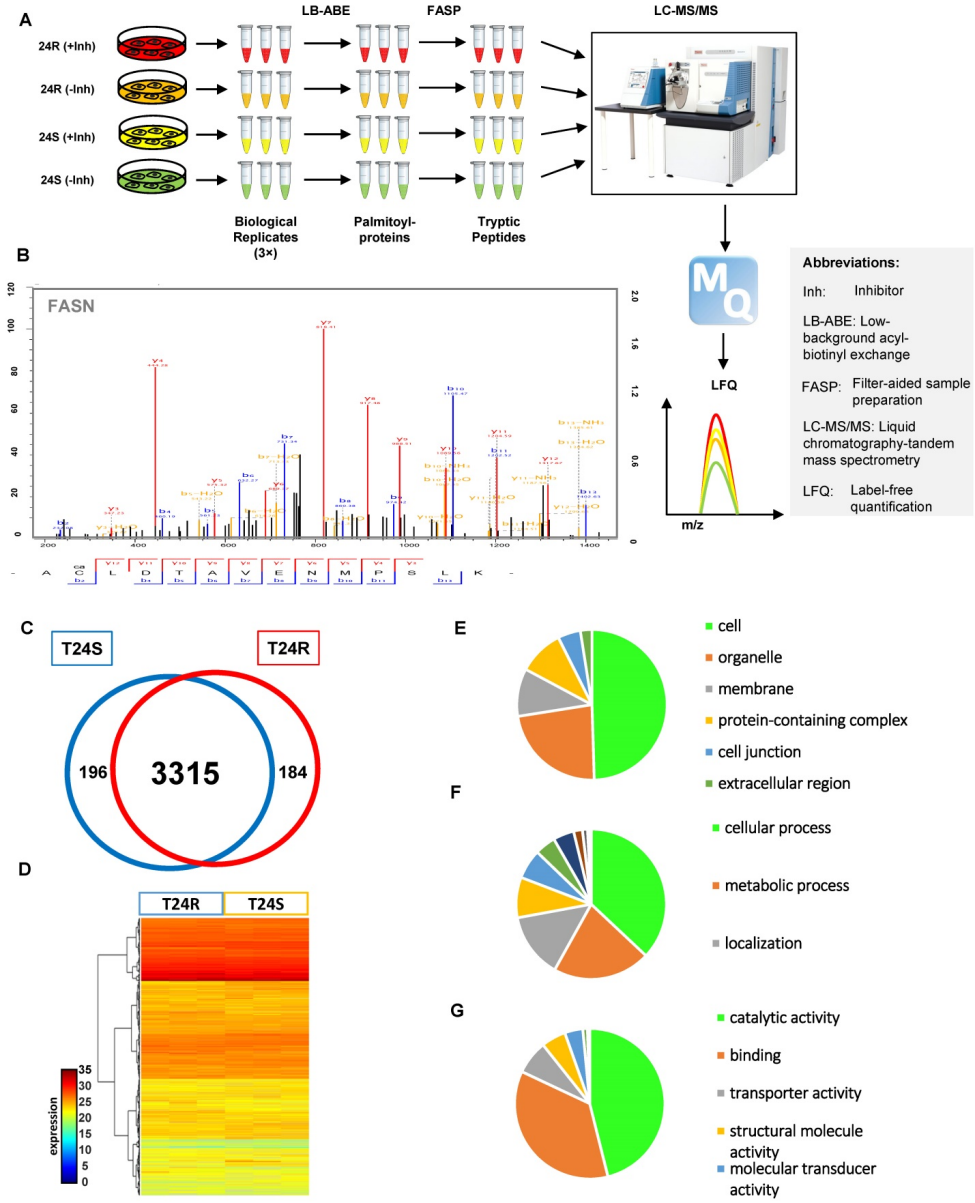
** (A)** Workflow for quantitative palmitoyl-proteomic comparison of two isogenic BC cells, T24R and T24S, using LFQ LC-MS/MS analysis following LB-ABE. After LB-ABE, a total of 16 samples (4 samples x 4 conditions) were digested in parallel into tryptic peptides by FASP, followed by LC-MS/MS. **(B)** Representative tandem mass spectrum of candidate palmitoyl peptide. LB-ABE-enriched proteins were separated by SDS-PAGE and digested in gel, followed by the extraction of tryptic peptides, which were analyzed by LC-MS/MS. Free cysteines in the purified peptides are candidate palmitoylation sites. **(C-G)** The detected palmitoylated proteins from T24R and T24S cells were classified by gene ontology categories. **(C)** Venn diagram shows the number of identified proteins in T24R and T24S cells. **(D)** Heatmap depicts abundance of commonly identified proteins in both T24R and T24S. Red and blue indicate high and low abundance of the proteins, respectively. **(E-G)** Pie chart visualizes the proportion of the number of proteins involved in the enriched gene ontology categories including cellular compartment **(E),** biological processes **(F),** and molecular functions **(G).**

Our group previously developed and characterized isogenic human BC cell lines, cisplatin sensitive (T24S) and resistant T24 BC cells (T24R) 51. In this study, we coupled LB-ABE enrichment with label-free proteomics to identify the potential link between protein palmitoylation and cisplatin resistance in BC. Briefly, we isolated palmitoyl-proteins from whole cell lysates by LB-ABE, digested them into peptides with trypsin using FASP, analyzed peptides by liquid chromatography-tandem mass spectrometry (LC-MS/MS), and performed database searching analysis and LFQ using MaxQuant. Our proteomics analysis workflow is summarized in **Figure 1A**. A total of 4,188 putative palmitoylated proteins were identified with a false discovery rate of ≤1%. Representative MS spectrum of FASN is shown in **Figure 1B**.

After filtering out the low-quality proteins, which are detected less than two replicates from T24S or T24R BC cells, we found that most of the palmitoylated proteins overlap in T24S and T24R cells (3,315 proteins: 89.71% of total) (**Figure 1C**). We also identified 184 proteins that were palmitoylated only in T24R cells, not in T24S, and 196 proteins that were palmitoylated only in T24S cells, not in T24R (**Figure 1C**). The heatmap shows that the palmitoylation levels of most proteins in T24S and T24R cells were not significantly different (**Figure 1D**).

**Supplementary table 1** shows the 3,695 palmitoylated proteins identified in both T24S and T24R BC cells. To assess the functions of these 3,695 palmitoylated proteins, we performed functional classification analysis using PANTHER 46. The location and function of these proteins are summarized in **Figures 1E-1G**. The identified palmitoylated proteins are functionally highly diverse; however, 35.1% of the classified proteins are related to metabolic processes and localization (**Figure 1F**).

**Figure 2:**
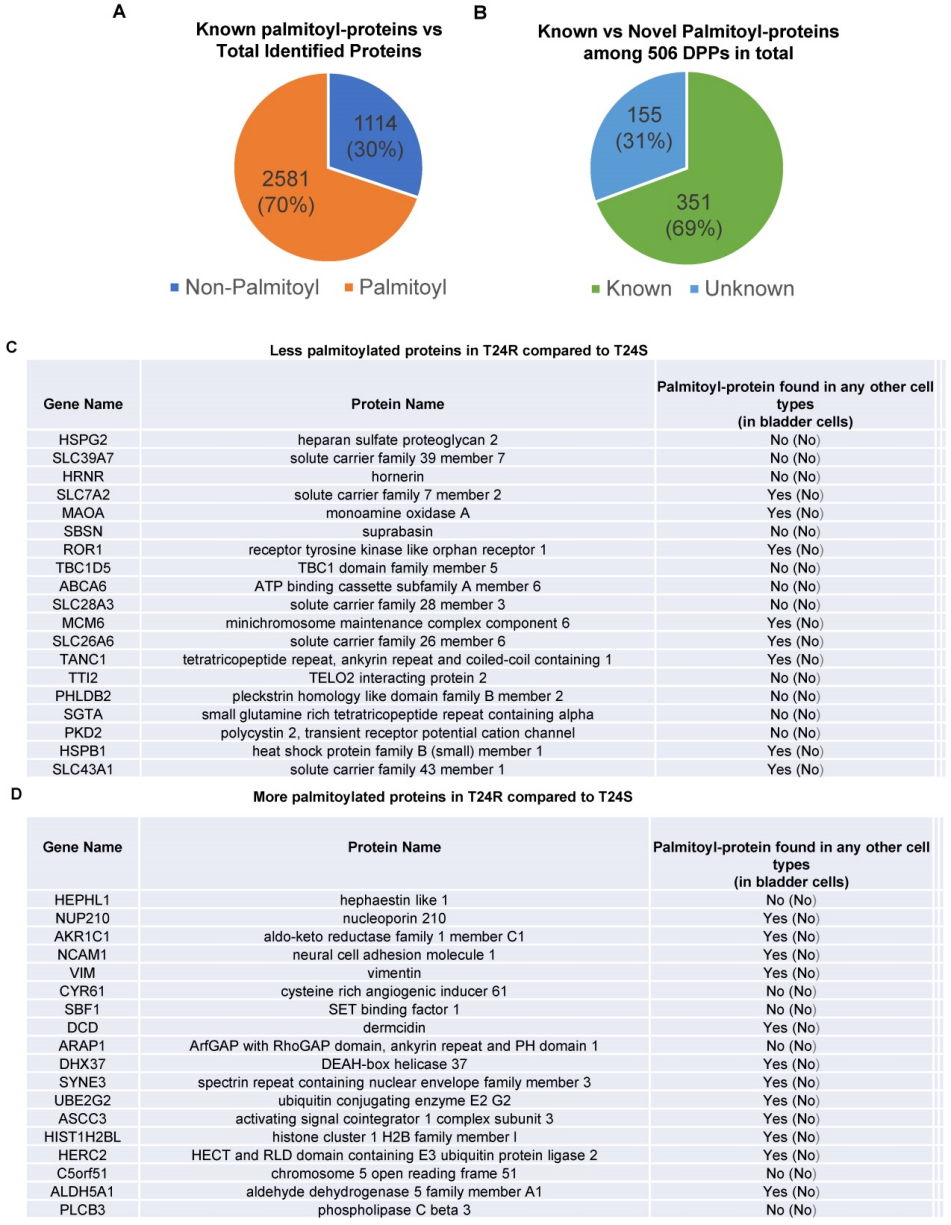
** Known and novel palmitoylated proteins from T24R and T24S cells. (A**) Pie chart depicts the proportion of palmitoylated and non-palmitoylated proteins from the total identified proteins. **(B)** Pie chart shows proportion of known and novel candidate palmitoyl-proteins. **(C-D)** Known and novel palmitoylated proteins in other cancer cells and bladder cells.

***DPPs between T24R and T24S cells.***Among the 3,695 identified proteins, at least 2,581 (70%) were previously reported to be palmitoylated in other human cells 29,52, confirming successful enrichment of palmitoyl-proteins in this study**.** Most of the 2,581 putative palmitoylated proteins were not reported in normal human bladder or BC cells (**Figure 2A**).

Next, we sought to identify DPPs in T24R cells compared to T24S. To further understand cisplatin resistance-associated protein palmitoylation changes, we aimed to identify DPPs between T24R and T24S cells. A statistical hypothesis test using an empirical null model was conducted (**see Methods**). From the total 3,315 putative palmitoylated proteins, 506 proteins were identified as DPPs based on the criteria of a combined P<0.05 and log2-transformed fold-change of 0.58 (**Figure 2B**). Furthermore, we found only 506 proteins as being differentially abundant in T24R cells compared to T24S after extensive statistical analysis. Among those identified 506 DPPs, at least 351 (69%) were identified as known palmitoyl proteins 29,52 (**Figure 2B**). To construct the database of known palmitoylated proteins, we combined the 3,593 human palmitoyl proteins compiled in the SwissPalm (v2), a compendium of palmitoyl proteins, with the 2,895 high-confidence candidate human palmitoyl proteins identified from LNCaP cells using LB-ABE to generate the currently most comprehensive palmitoyl-proteome database, containing 4,669 known palmitoyl proteins. Then, by comparing the 506 differentially abundant palmitoyl proteins with the 4,669 known human palmitoyl proteins, 351 DPPs were identified as known palmitoyl-proteins. The remaining 155 (31%) DPPs are novel putative palmitoyl-proteins and not reported in any type of cells (**Figure 2B**). A few examples of known and novel DPPs were shown in **Figures 2C and 2D**.

### Cisplatin resistance-associated protein palmitoylation changes

**Figure 3:**
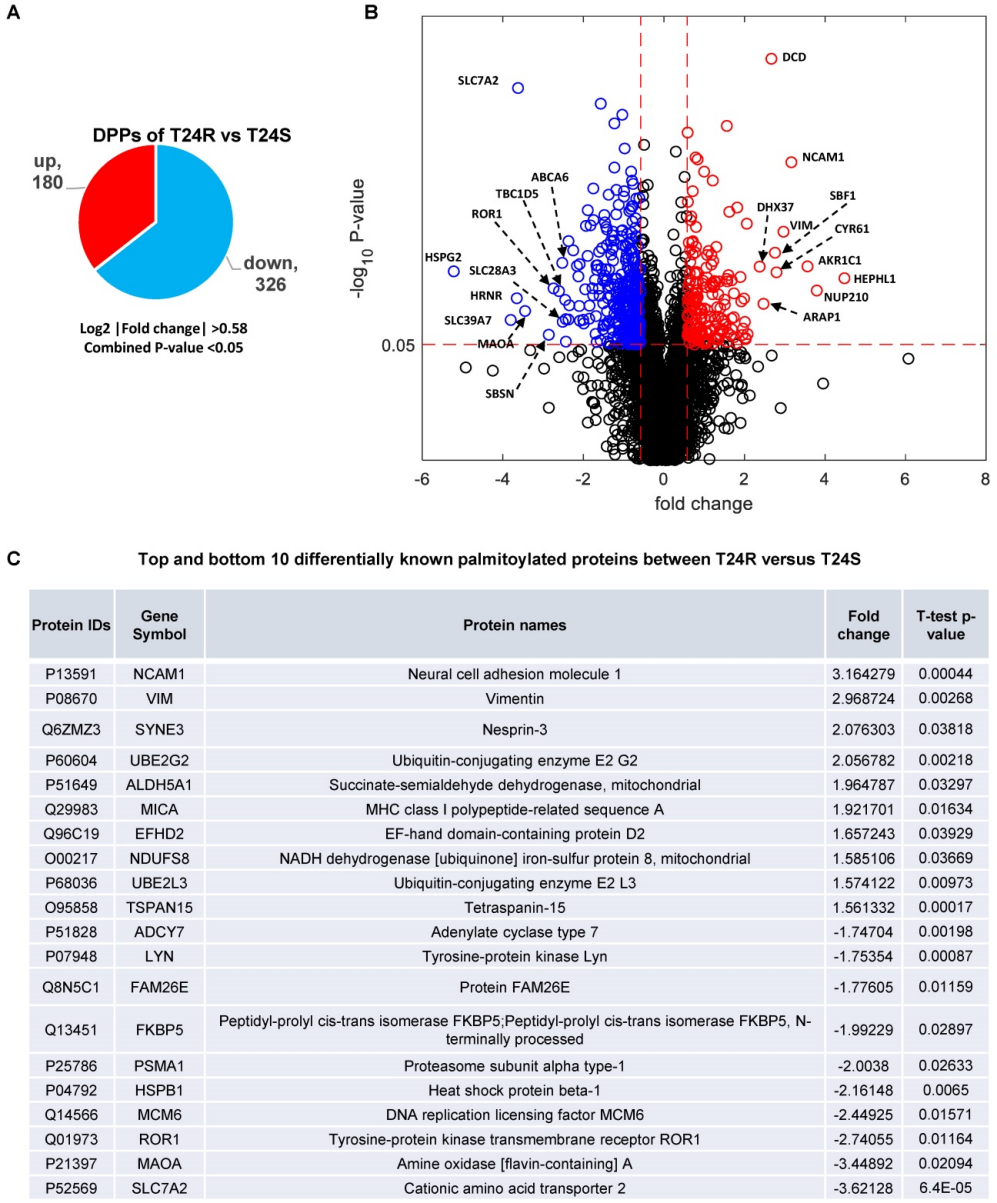
** Identification of differentially palmitoylated proteins (DPPs).** (**A**) Diagram showing 180 up and 326 downregulated DPPs in T24R cells. (**B**) Volcano plot shows all putative palmitoylated proteins. Red, upregulated DPPs in T24R cells compared to T24S (n=180). Blue, downregulated DPPs in T24R cells compared to T24S (n=326).** (C)** Top 10 most and least DDPs among known palmitoylated proteins.

These 506 DDPs included 180 upregulated and 326 downregulated DPPs in T24R cells, compared to T24S **(Figure 3A)**. A volcano plot displays DPPs between the T24R vs. T24S cells (**Figure 3B**). Among these DDPs, NCAM1, VIM, ROR1, MAOA, and SLC7A2 are known to be palmitoylated in other types of cells^52^. **Figure 3C** summarizes the most altered known palmitoylated proteins in T24R compared to T24S cells.

### Biological and mechanistic meaning of DDPs associated with cisplatin resistance

**Figure 4:**
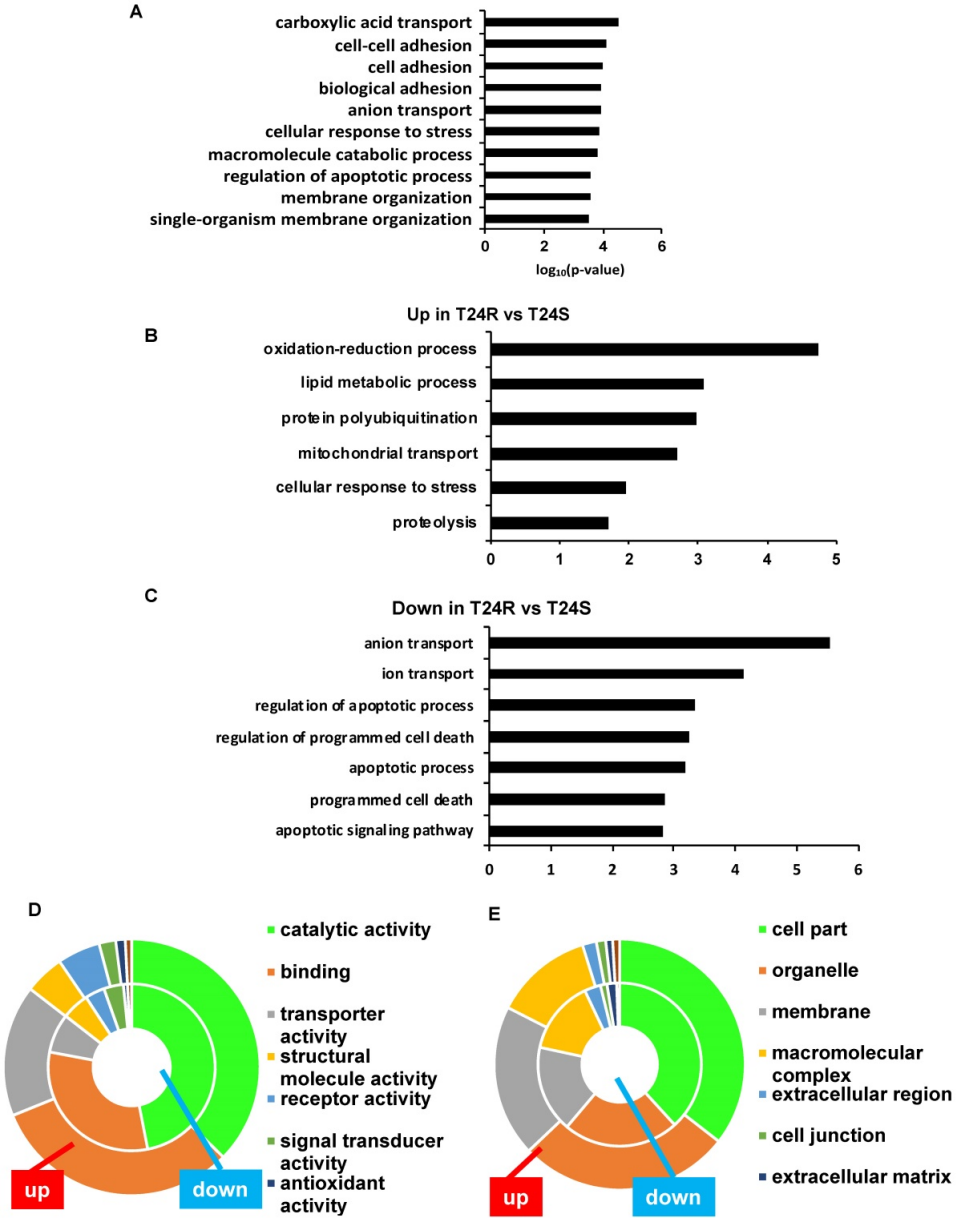
** Biological enrichment of DPPs between T24R and T24S cells. (A)** Bar graph shows top 10 biological processes enriched by DPPs in T24R cells compared to T24S. (**B-C**) Bar graphs show enriched biological processes upregulated **(B)** and downregulated by DPPs in T24R cells **(C). (D-E)** Doughnut charts visualize the proportion of the numbers of up (outside donut) and downregulated (inside donut) DPPs in the enriched molecular functions (**D**) and cellular compartments (**E**)**.**

The top 10 significantly enriched biological processes suggest that carboxylic acid transport, cell-cell adhesion, and biological adhesion were enriched for by DPPs in T24R cells (**Figure 4A**). Further functional enrichment analysis of the 180 upregulated DDPs in T24R cells revealed that oxidation-reduction or lipid metabolic processes were enriched, while the 326 downregulated DDPs were enriched for anion/ion transport and apoptosis (5 out of top 7 biological processes) (**Figure 4B**)**.** The following doughnut charts exhibit the proportion of the numbers of up (outside) and downregulated DPPs (inside) for enriched molecular functions (**Figure 3B**) and cellular compartments (**Figure 4C**)**.** In terms of molecular functions, those related to catalytic activity or binding were enriched for in both up and downregulated DDPs, suggesting that palmitoylation functions in membrane anchoring, trafficking, and cellular localization-associated enzymatic activity (**Figure 4D**)**.** Cellular compartments, such as the organelles and membrane, were also enriched for by DDPs (**Figure 4E**)**.**

**Table 1** shows the enriched biological processes of DPPs between T24R and T24S cells. Functional analysis suggested that proteins related to oxidation-reduction and lipid metabolism pathways are highly palmitoylated in T24R cells. In contrast, those related to ion and anion transport were significantly downregulated in T24R cells. Collectively, our data imply that protein palmitoylation may be involved in a wide range of biological processes and aggressiveness of cancer, which can be further associated with cisplatin resistance.

### Inhibition of palmitoylation differently perturbed protein palmitoylation in T24S and T24R cells

**Figure 5:**
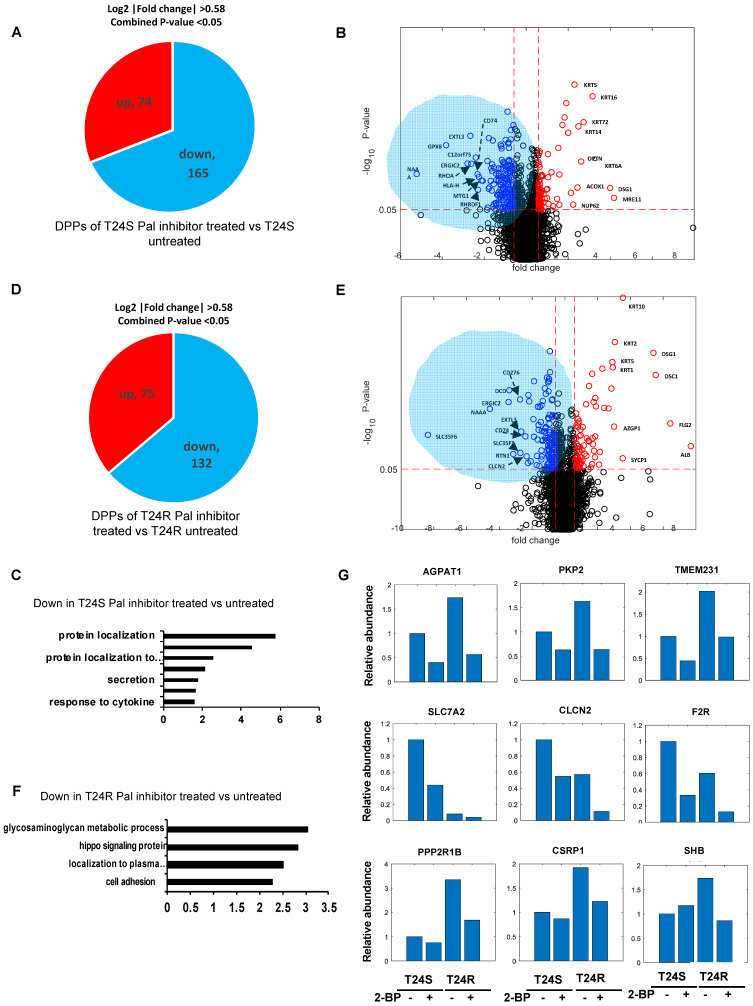
** Effects of palmitoylation inhibition in T24S and T24R cells (A)** Pie chart depicts the number of up and downregulated DPPs by palmitoylation inhibitor treatment in T24S cells. **(B)** Volcano plot shows the distribution of the DPPs with log2 ratio of palmitoylation inhibitor treated and untreated T24S cells (x-axis) and statistical significance (y-axis). **(C)** Bar plot shows downregulated biological processes in T24S cells in response to the inhibitor treatment**. (D)** Pie chart depicts the number of up and downregulated DPPs by palmitoylation inhibitor treatment in T24R cells**. (E)** Volcano plot shows the distribution of the DPPs with log2 ratio of inhibitor treated and untreated T24R cells (x-axis) and statistical significance (y-axis). **(F)** Bar plot shows downregulated biological processes in T24R cells in response to the inhibitor treatment. **(G)** Palmitoylated protein abundance with or without palmitoylation inhibitor in T24S and T24R cells. Examples of palmitoyl-proteins that have different effects on the treatment of palmitoylation inhibitor are shown. (**Top**) Three proteins exhibit higher expression in T24R cells compared to T24S and have significant downregulation after inhibitor treatment in both. (**Middle**) Three proteins are significantly higher in T24S cells compared to T24R and have significant downregulation in both. (**Bottom**) Three proteins are significantly higher in T24R cells compared to T24S and have significant downregulation only in T24R.

A lipid-based protein palmitoylation inhibitor, 2-BP, was used to test how palmitoylation of identified DPPs are regulated in T24R and T24S cells. In T24S cells, 165 DDPs were identified as being downregulated in response to 2-BP (log2-fold-change >0.58, combined p-value <0.05) (**Figure 5A**). A volcano plot exhibits the DDPs significantly altered by palmitoylation inhibition (**Figure 5B**). The enriched biological processes among downregulated DDPs indicated protein localization (**Figure 5C**), while those upregulated include intermediate filament, skin development, and fatty acid beta-oxidation. (**Figure S1A**).

In T24R cells, 75 DDPs were upregulated and 132 were downregulated by palmitoylation inhibition (log2-fold-change >0.58, combined p-values <0.05) (**Figure 5D**). A volcano plot exhibits up or downregulated DDPs after 2-BP treatment (**Figure 5E**). The enriched biological processes among the 132 processes downregulated by palmitoylation inhibition indicated the glycosaminoglycan metabolomic process, hippo signaling, and protein localization to the plasma membrane (**Figure 5F**); while those upregulated include epidermis development, skin development, cell junction organization, and fatty acid beta-oxidation. (**Figure S1B**).

There were several clustered patterns of palmitoylation changes among DPPs. For example, palmitoylation levels of DDPs, such as AGPAT1 (1- acylglycerol-3-phosphate O-acyltransferase 1), PKP2 (plakophilin 2), TMEM231 (transmembrane protein 231), SLC7A2 (solute carrier family 7 member 2), CLCN2 (chloride voltage-gated channel 2), and F2R (protease-activated receptors), decreased in both T24S and T24R cells after 2-BP treatment (**Figure 5G**). In contrast, DPPs, like PPP2R1B (protein phosphatase 2 scaffold subunit A beta), CSRP1 (cysteine and glycine rich protein 1), SHB (SH2 domain containing adaptor protein B), were decreased only in T24R cells following 2-BP treatment (**Figure 5G**). Collectively, these findings suggest that a variety of palmitoylation mechanisms may play a role in the regulation of protein palmitoylation. In both T24S and T24R cells, there were noticeable common biological processes affected by palmitoylation inhibition, including protein localization and fatty acid beta-oxidation.

### ACSS2 inhibition decreases fatty acid synthesis and changes palmitoylation of proteins in BC cells

**Figure 6:**
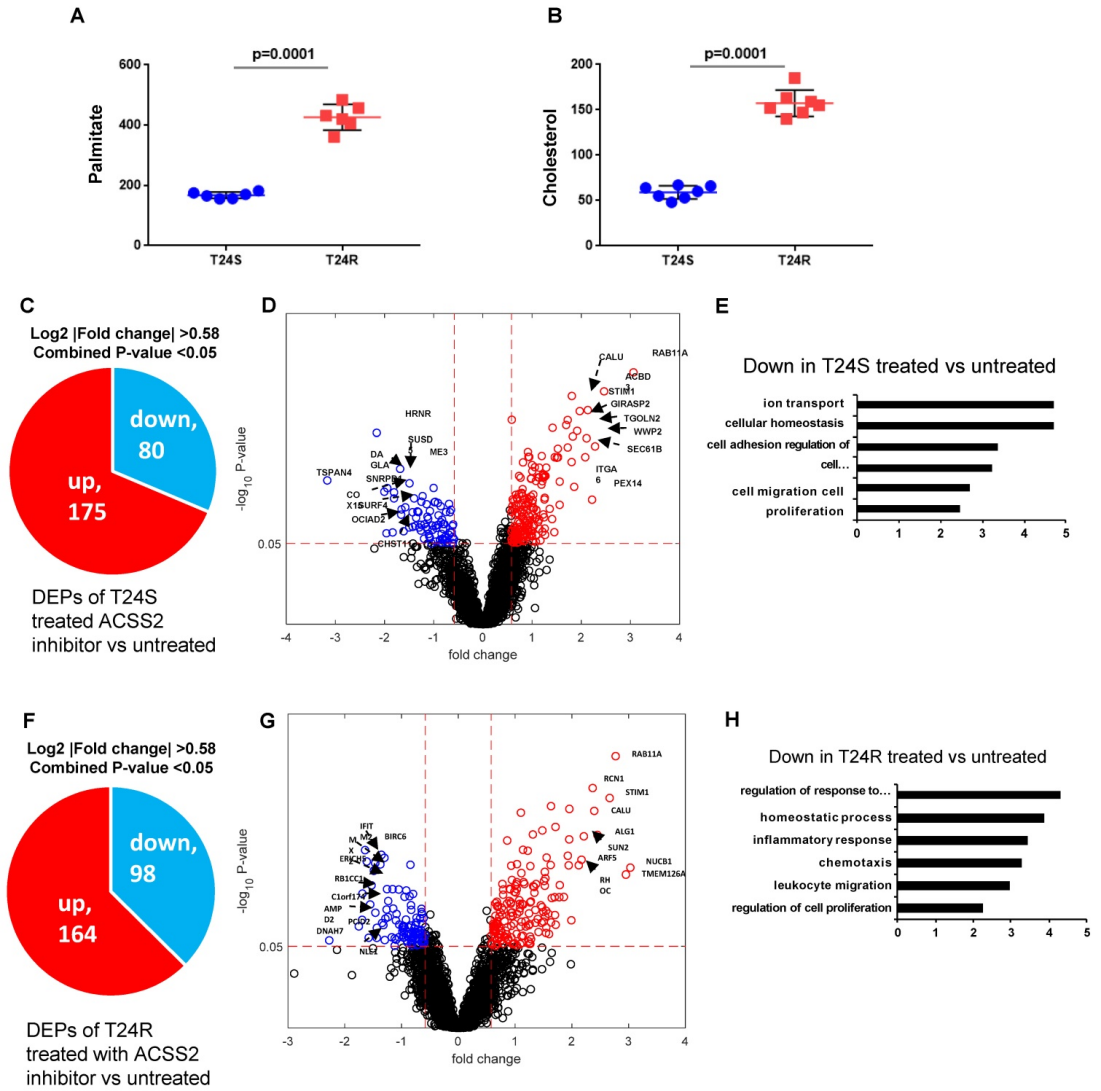
** Effects of ACSS2 inhibitor in T24S and T24R. (A)** Palmitate concentration in T24S and T24R cells. **(B)** Cholesterol levels were compared in T24S and T24R cells. **(C)** Pie chart depicts the number of up (n=175) and downregulated DPPs (n=80) following ACSS2 inhibitor treatment in T24S cells. **(D)** Volcano plot shows the distribution of the DPPs with log2 ratio of ACSS2 inhibitor treated and untreated T24S cells (x-axis) and statistical significance (y-axis). **(E)** Bar plot shows downregulated biological processes in T24S cells in response to ACSS2 inhibitor treatment. **(F)** Pie chart depicts the number of up (n=164) and downregulated DPPs (n=98) following ACSS2 inhibitor treatment in T24R cells. **(G)** Volcano plot shows the distribution of the DPPs with log2 ratio of ACSS2 inhibitor treated and untreated T24R cells (x-axis) and statistical significance (y-axis). **(H)** Bar plot shows downregulated biological processes in T24R cells in response to ACSS2 inhibitor treatment.

Our previous study extensively investigated and demonstrated the link between lipid production and cisplatin resistance. The total lipid levels in T24R cells were significantly higher than those in T24S cells (~170%) 52. In T24R cells, expression of several lipid metabolism-related proteins, such as ACC (acetyl-CoA carboxylase), FASN, and ACSS2 (acyl-CoA synthetase short chain family member 2), was found to be increased. Since FASN is a key player in palmitate synthesis, the influence of palmitate levels and cholesterol concentration was examined in the context of cisplatin resistance. Experimental results from our previous 53 and current study show increased palmitate, cholesterol, and lipid production in T24R cells compared to T24S (**Figure 6A and 6B**). Our group previously found that ACSS2 inhibition decreased the *de novo* synthesis of fatty acid by more than 60% in T24R cells, but not in T24S 51.

Given these findings, we speculated that increased palmitate and cholesterol production via ACSS2 in T24R promotes changes in palmitoylation status of specific proteins; thereby, contributing to cisplatin resistance. An LC-MS/MS approach was used to determine if ACSS2 inhibition perturbs palmitoylation status of proteins in T24S and T24R cells. In T24S cells, bioinformatic analysis revealed 255 DPPs, of which 80 were upregulated and 175 were downregulated (**Figure 6C**). The volcano plot in **Figure 6D** shows the downregulated proteins and their palmitoylation levels following ACSS2 inhibition treatment. The biological processes of these DPPs include ion transport, cellular homeostasis, cell adhesion, cell migration, and proliferation (**Figure 6E**). In T24R cells, ACSS2 inhibition led to 98 downregulated DDPs (**Figure 6F and 6G**). The downregulated DDPs are involved in immune response related biological processes, such as regulation of response to external stimulus, homeostatic process, inflammatory response, chemotaxis, leukocyte migration, and regulation of cell proliferation (**Figure 6H**).

### FASN palmitoylation and PD-L1 expression in BC

The palmitoylation of FASN was first validated because its link to lipid metabolism is well established in many cancer types, including BC 33,36,54,55. FASN was found to be more palmitoylated in T24R cells compared to T24S. Because palmitoylation often contributes to increased protein stability and eventual upregulated expression, FASN expression could be associated with BC cisplatin resistance. We next tested a possible link between increased lipid production and palmitoylation with cisplatin resistance in BC.

**Figure 7:**
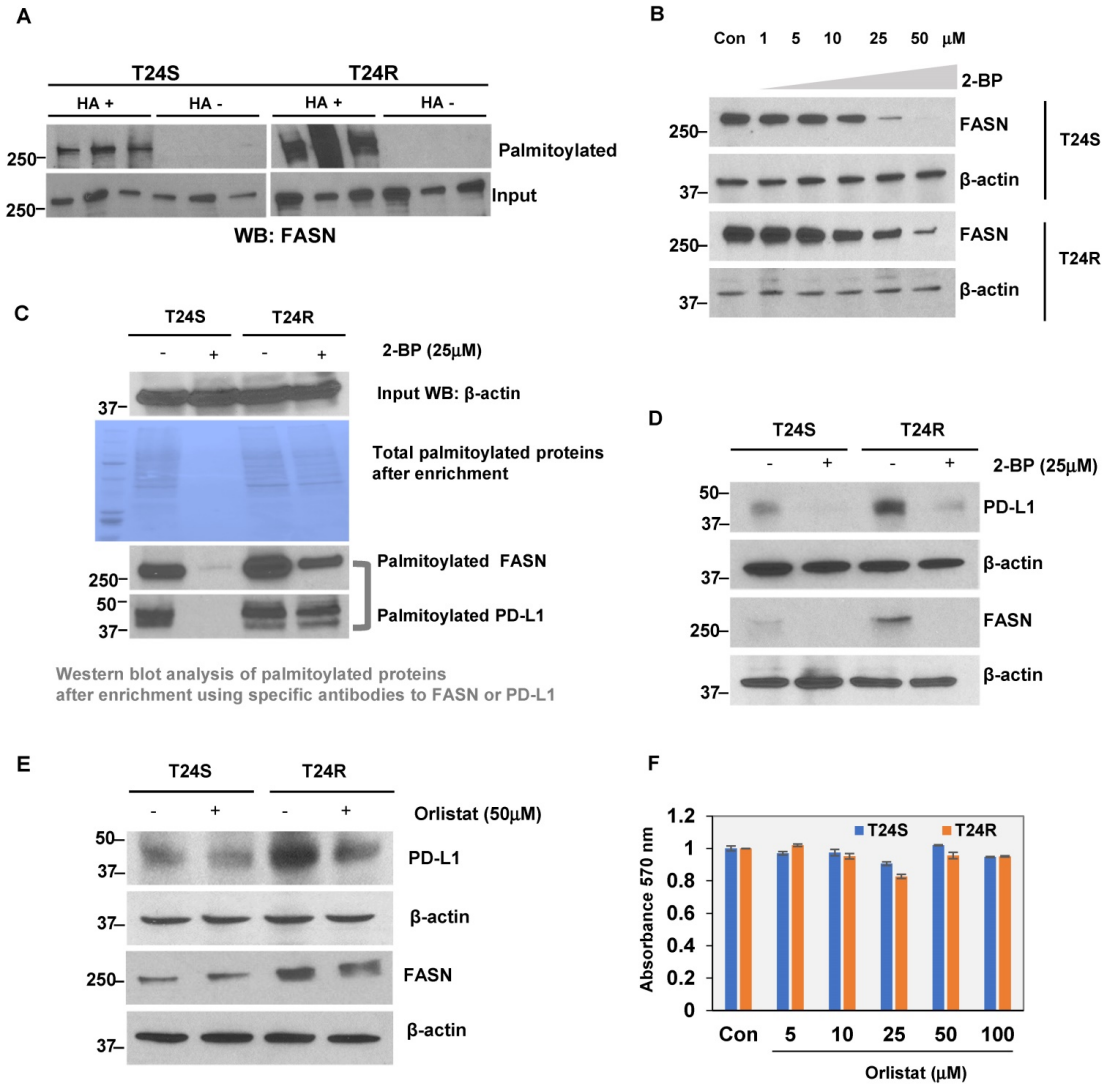
** (A)** Validation of FASN palmitoylation in T24S and T24R cells. Western blot analysis was performed using palmitoylation-enriched proteins. The same amount of proteins was used as starting materials (input). **(B)** FASN protein expression is downregulated by palmitoylation inhibition. Vehicle-treated cells acted as controls. **(C)** Further validation of FASN palmitoylation using a commercial kit. PD-L1 was also palmitoylated both in T24S and T24R cells.** (D)** Palmitoylation inhibition by 25 μM 2-BP treatment dramatically reduced protein expression of PD-L1 and FASN. Western blot analysis of FASN and PD-L1 protein expression from cells exposed to either 2-BP or vehicle control. **(E)** Expression of PD-L1 decreased in response to 50 μM orlistat treatment.** (F)** Cell viability assays on the effects of orlistat at varying time points or concentrations did not show any significant difference. Differences in cell viability, in which vehicle acted as control, was determined by t-test. Data are representative of at least three different experiments. Error bars denote SEM. *, **, and n.s. stand for *p* < 0.01, *p* < 0.05, and *p* ≥ 0.05, respectively.

At baseline, T24R cells were found to express modest but greater levels of FASN expression than T24S (**Supplementary Figure 2**). The LB-ABE method was used to validate whether FASN was palmitoylated in BC cells. FASN palmitoylation levels were increased in T24R cells compared to T24S (**Figure 7A**). There were more palmitoylated FASN proteins detected as well. Palmitoylation inhibition by 2-BP repressed FASN expression in a dose-dependent manner in both T24S and T24R cells (**Figure 7B**). This implies that FASN is palmitoylated in BC cells, and the increase in palmitoylation of FASN is likely associated with cisplatin resistance in BC.

Additional experiments were conducted to support the FASN palmitoylation data using commercial palmitoylation protein assay kits, as described in the **Methods**. These kits are based on similar enrichment principles as our own. After palmitoylation enrichment, the total palmitoylation levels were almost identical in T24R and T24S cells. However, after treatment with 2-BP, the total palmitoylated proteins were significantly reduced in T24S cells compared to T24R, which had partial remaining palmitoylated proteins. Further data using western blot analysis showed that palmitoylated FASN was increased in T24R cells compared to T24S (**Figure 7C, palmitoylated FASN panel**). PD-L1 was also found to be palmitoylated in both T24S and T24R cells. Palmitoylation levels of PD-L1 were greater in T24R cells with or without 2-BP treatment (**Figure 7C, palmitoylated PD-L1 panel**). PD-L1 expression was determined to be approximately 15-fold greater in T24R cells compared to T24S (**Figure 7D**). When palmitoylation was inhibited by 2-BP, PD-L1 expression was significantly reduced in both T24R and T24S cells (**Figure 7D**). Although 2-BP abolished PD-L1 palmitoylation completely, it was not able to effectively suppress PD-L1 palmitoylation in T24R cells.

Next, the effects of FASN inhibition on BC-specific palmitoylated proteins was examined. T24S and T24R cells were treated with orlistat. This reduced PD-L1 protein expression in both T24R and T24S cells (**Figure 7E**), suggesting that FASN activity likely regulates PD-L1 palmitoylation and stability. It was confirmed that there were no cytotoxic effects of orlistat within the conditions used, based on additional analysis showing that there were no observed changes in cell viability in response to orlistat in both T24S and T24R cells (**Figure 7F**).

## Discussion

S-palmitoylation (S-acylation) is the enzymatic addition of a fatty acid (acyl) group, such as palmitate, onto cysteine residues of a protein via thioester linkage, which is catalyzed by palmitoyltransferases and depalmitoyltransferases 56. Many protein substrates can be palmitoylated by more than one DHHC enzyme with certain DHHC-substrate specificity. DHHCs may act as a functional heterodimer, which may affect their enzymatic activities. *S*-palmitoylation is a powerful regulatory mechanism for a number of cellular processes, including signal transduction, protein turnover, vesicle fusion, and cell-cell interactions. Dysfunctions can lead to cancer, cardiovascular disease, and neurological disorders 57-59. The reversible modification of cysteine residues by thioester formation with palmitate by the addition of a C16:0 carbon palmitoyl moiety is an abundant lipid post-translational modification. This addition of palmitate enhances a protein's affinity to the membrane, directs its distribution in membrane micro-domains, and mediates protein-protein interactions, trafficking, stability, and aggregation state. There is an intriguing potential connection between alterations in the metabolome and mitochondrial regulation. In addition, post-translational lipid modification targets and shuttles proteins between the cytosol and lipid raft within the plasma and mitochondrial membranes. Palmitoylation is known to play active roles in the sorting and trafficking of many proteins, and fluctuations in palmitoylation may contribute to signaling outcomes. S-palmitoylation has been observed in many ER and mitochondrial proteins, suggesting an intriguing potential connection between metabolic lipids and mitochondrial regulation 60. However, it is unknown whether or how mitochondrial S-palmitoylation is regulated in the context of resistance against chemotherapy 16,61-63.

Our palmitoyl-proteomics approach detected a total of 25,598 peptides and 3,695 putative palmitoylated proteins in either T24S or T24R BC cells. The further defined 506 DDPs included 180 upregulated and 326 downregulated palmitoylated proteins in T24R cells. Our results also uncovered a novel molecular mechanism of palmitoylation, which demonstrates that protein palmitoylation of putative candidate proteins, is linked with responsiveness to chemotherapy, such as cisplatin. In this study, our palmitoylated protein-enriched proteomics profiling comparing cisplatin-resistant and sensitive BC cells showed that FASN is critical for protein palmitoylation in cisplatin-resistant cells. FASN plays an important role in synthesis of palmitate, which is both a precursor for fatty acids and the acyl group that is added to cysteine residues during palmitoylation 64. Therefore, it is logical to infer that overexpression of FASN is associated with increased protein palmitoylation, which then contributes to worse prognoses in certain cancers 65. Furthermore, a recent study found that FASN mediates EGFR palmitoylation in EGFR-mutated chemo-resistant non-small cell lung cancer 32.

We found that FASN expression and palmitoylation may lead to increased PD-1 expression and palmitoylation. PD-L1, a T cell regulatory molecule that is expressed on the surface of tumor and tumor-infiltrating immune cells, was also found to be highly palmitoylated in cisplatin-resistant BC cells, compared to isogenic control cells 66. Activation of the pathway inhibits the activation of cytotoxic T lymphocytes and is one of the main methods through which tumor cells evade immune responses 67. Antibodies targeting PD-L1 are known to benefit overall survival in BC, and several agents have received accelerated approval from the FDA for treatment 68. In addition to being a clinical target, PD-L1 has also been shown to be a significant clinical predictor for stage and treatment response in BC 69. It has been previously shown that PD-L1 is palmitoylated via a covalent attachment of palmitic acid to its 272 cysteine residue for stability 70. Although the effects of PD-L1 palmitoylation has not been thoroughly examined in the context of BC, previous studies have shown that palmitoylation stabilizes PD-L1 and promotes tumor growth in other cancer types, such as breast cancer 71, melanoma, and BC. Based on this prior evidence, exploring the influence of PD-L1 palmitoylation in BC progression and aggression presents a promising opportunity.

There are limitations in this study. Although there are ongoing studies in our laboratory, this study did not provide evidence to demonstrate whether inhibition of both FASN and PD-L1 may have synergistic inhibitory effects on cancer progression. These alternative hypotheses await further investigation. Currently, the most widely used palmitoylation inhibitor, 2-BP, a non-metabolizable palmitate analog, elicits pleiotropic effects. However, no inhibitory drugs targeting palmitoylation with high-affinity and specificity are available. There is an urgent need to identify specific, high-affinity inhibitors of protein palmitoylation for basic research and therapeutic intervention based on palmitoylation. Thus, much work remains to be developed on the specific and high affinity inhibitors of protein palmitoylation that can be applied as therapeutic strategies designed for overcoming cisplatin resistance in BC. In this study, we did not focus on the specific enzymes that catalyze palmitoylation of PD-L1. Further analysis on how palmitoylases and depalmitoylases, such as ZDHHC and APT/PPT, control PD-L1 palmitoylation and activity and contribute to activation of oncogenic pathways is warranted. Additional exploration of these mechanisms is necessary to clarify how PD-L1 is trafficked in the cell and how its activity is controlled in the context of cisplatin resistance.

## Supplementary Material

Supplementary figures and tables.

## Figures and Tables

**Table 1:** Enriched biological process of DPPs

	GOBPs	Gene count	P-value	Genes
T24R vs T24S Up	cell adhesion	29	0.006294	ARHGAP5, ATP1B1, CASP8, CSRP1, DSP, CYR61, LDHA, BCAM, ME1, NCAM1, NEO1, PKP2, PLCB3, PLXNB3, PSMB10, SHB, SHC1, STK10, TPBG, SCARF1, NRP2, ME3, TMOD3, OLA1, ERBIN, ESYT2, EFHD2, VASN, MICA.
	lipid metabolic process	27	0.000834	ACAA1, ACACA, ALDH1A3, ALDH3A2, CREBBP, AKR1C1, DHCR7, GM2A, HMGCS1, CYR61, PITPNA, PLCB3, ABCD3, SCD, ALDH5A1, SCARF1, ACAA2, AGPAT1, DDX20, PTGR1, ACSL5, NANS, DOLPP1, MBOAT7, PTPMT1, CERS6, IAH1.
	oxidation-reduction process	26	0.000018	ACAA1, ALDH1A3, ALDH3A2, ALDOC, COX15, AKR1C1, DHCR7, LDHA, ME1, NDUFS8, PGD, ABCD3, PYGB, SCD, SPR, ALDH5A1, ACAA2, ME3, PTGR1, PRDX5, KDM3A, SQOR, NXN, VKORC1L1, QSOX2, HEPHL1.
T24R vs T24S Down	cell adhesion	45	0.005795	CD59, CLPTM1, COL6A1, DNM2, DSC3, HDLBP, HSPB1, JUP, LAMC1, LGALS1, LRP6, LYN, TACSTD2, MCAM, NCAM2, PDPK1, PNN, PODXL, PRKCD, PSEN1, RANGAP1, ROBO1, RPL22, S100A11, SLC9A1, ZEB1, TGFB1, TGFBR2, MAD1L1, RIPK2, TJP2, FLOT1, PACSIN2, CLASP2, MPRIP, ICOSLG, NECTIN3, TES, BAIAP2L1, JAM2, EPS8L2, VMP1, CD99L2, ANTXR1, PHLDB2.
	ion transport	47	0.000072	ABCD1, CLCN2, CLCN7, CLN3, COX4I1, DNM2, DPYSL2, STOM, F2R, GLS, LYN, ABCC1, P2RX4, PDPK1, PKD2, PLP2, PSEN1, PSEN2, SLC6A9, SLC7A2, SLC9A1, SLC12A2, SLC25A1, SNAP25, TGFB1, SLC39A7, SLC43A1, SLC5A6, SLC16A3, TMEM63A, SLC12A7, SERINC3, SLC39A14, CALHM2, TRPV2, SLC38A2, MCOLN1, SERINC1, SLC26A6, TTYH3, SLC38A1, MCU, ORAI3, SLC46A1, NIPA1, SLC9B2, CALHM5.
	anion transport	25	0.000003	ABCD1, CLCN2, CLCN7, CLN3, DPYSL2, GLS, ABCC1, P2RX4, PSEN1, SLC6A9, SLC7A2, SLC12A2, SLC25A1, SNAP25, SLC43A1, SLC5A6, SLC16A3, SLC12A7, SERINC3, SLC38A2, SERINC1, SLC26A6, TTYH3, SLC38A1, SLC46A1.
