# Supplementary material for: S-Palmitoylation as a Functional Regulator of Proteins Associated with Cisplatin Resistance in Bladder Cancer
Source: Int J Biol Sci. 2020 Jul 19;16(14):2490–505. doi: 10.7150/ijbs.45640 (PMC7415425; doi:10.7150/ijbs.45640)

SUPPLEMENTARY FIGURE LEGENDS

Figure S1. (**A**) Bar graphs show enriched biological processes by downregulated DPPs in T24R cells**. (B)** Bar plot shows upregulated biological processes in T24R cells in response to inhibitor treatment. (**C**) Three proteins are significantly higher in T24S cells compared to T24R and have significant downregulation only in T24R cells after inhibitor treatment. (**D**) Three proteins are significantly higher in T24R cells compared to T24S and inhibitor treatment renders significant upregulation of proteins only in T24R cells. (**E**) Three proteins are significantly higher in T24S cells compared to T24R and the inhibitor renders significant upregulation of the proteins only in T24R.

Figure S2. Expression of FASN in T24S and T24R cell lines.

Supplementary Figure 1.

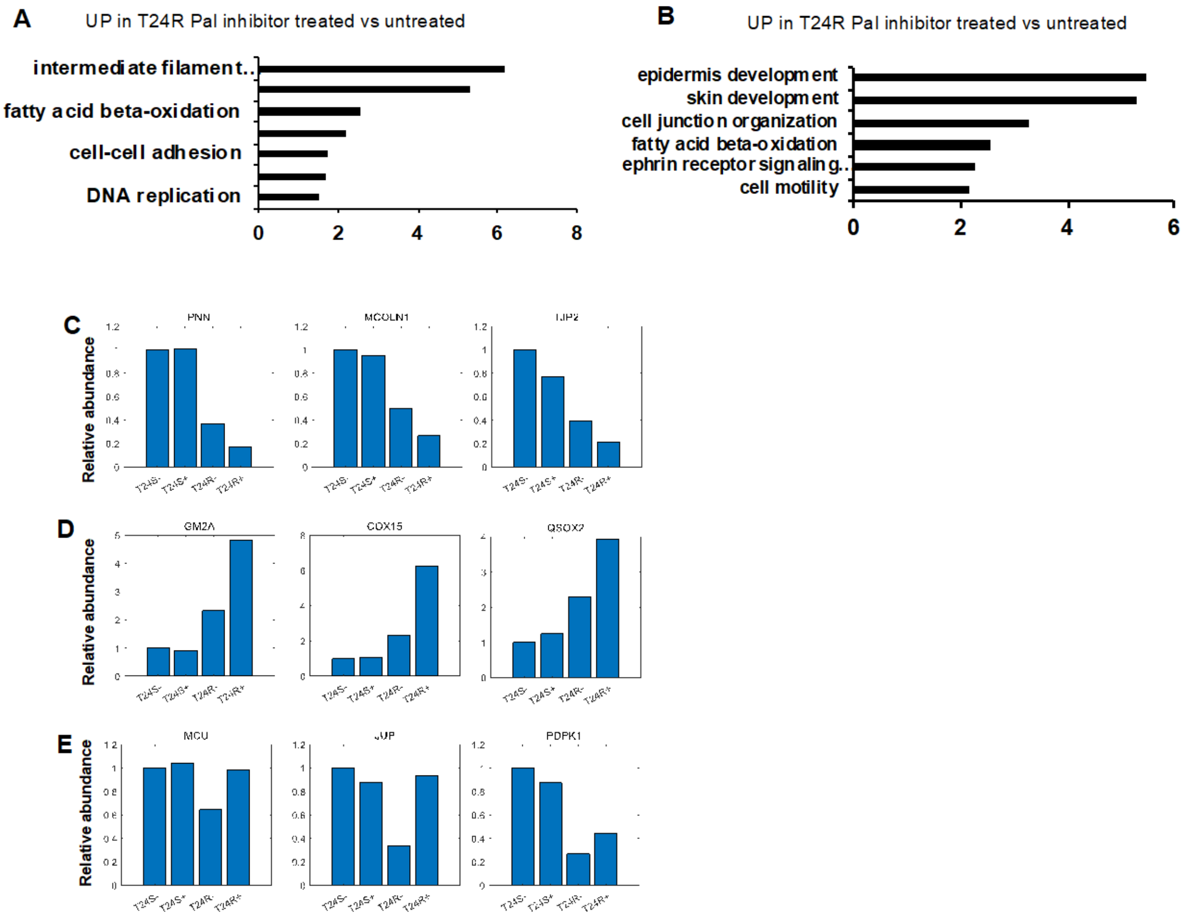

Supplementary Figure 2.

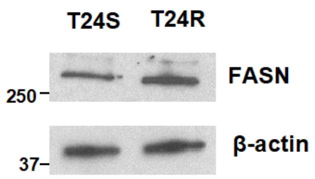

Supplement: Supplementary file 1 — Supplementary figures and tables. [file ijbsv16p2490s1.pdf]
